# Positron Emission Computed Tomography Imaging of Synaptic Vesicle Glycoprotein 2A in Alzheimer’s Disease

**DOI:** 10.3389/fnagi.2021.731114

**Published:** 2021-11-02

**Authors:** Yanyan Kong, Shibo Zhang, Lin Huang, Chencheng Zhang, Fang Xie, Zhengwei Zhang, Qi Huang, Donglang Jiang, Junpeng Li, Weiyan Zhou, Tao Hua, Bomin Sun, Jiao Wang, Yihui Guan

**Affiliations:** ^1^PET Center, Huashan Hospital, Fudan University, Shanghai, China; ^2^Laboratory of Molecular Neural Biology, School of Life Sciences, Shanghai University, Shanghai, China; ^3^Department of Neurosurgery, Ruijin Hospital, Shanghai Jiao Tong University School of Medicine, Shanghai, China

**Keywords:** Alzheimer’s disease, molecular imaging, synaptic vesicle glycoprotein 2A, early diagnosis, synapses

## Abstract

Alzheimer’s disease (AD) is the most common neurodegenerative disorder seen in age-dependent dementia. There is currently no effective treatment for AD, which may be attributed in part to lack of a clear underlying mechanism. Early diagnosis of AD is of great significance to control the development of the disease. Synaptic loss is an important pathology in the early stage of AD, therefore the measurement of synaptic density using molecular imaging technology may be an effective way to early diagnosis of AD. Synaptic vesicle glycoprotein 2A (SV2A) is located in the presynaptic vesicle membrane of virtually all synapses. SV2A Positron Emission Computed Tomography (PET) could provide a way to measure synaptic density quantitatively in living humans and to track changes in synaptic density in AD. In view of the fact that synaptic loss is the pathology of both epilepsy and AD, this review summarizes the potential role of SV2A in the pathogenesis of AD, and suggests that SV2A should be used as an important target molecule of PET imaging agent for the early diagnosis of AD.

## Introduction

The most prominent pathophysiological features of Alzheimer’s disease (AD) include the aggregation of intracerebral β-amyloid (Aβ), excessive phosphorylation of tau protein, and hypofunction and loss of neurons and synapses, which predate the onset of the clinical symptoms of AD. Effective treatments for AD are still lacking, and this is primarily due to difficulties in the early diagnosis of AD and the late treatment of patients. Recent research results have brought hope to people in the treatment of early AD, but there is still a lack of effective diagnosis or treatment in clinical practice (Siemers et al., 2016; Schwarz et al., 2019). However, there are still challenges in the early diagnosis of AD. Sebastian Palmqvist et al. (2020) used the plasma phospho-tau217 (P-tau217) as a biomarker in the diagnosis of neurodegenerative diseases. The accuracy of the diagnosis of P-tau217 is higher than other biomarkers in plasma, but its clinical application still needs to be further optimized (Palmqvist et al., 2020). Identifying the early diagnostic markers from the pathophysiological features of AD and its preceding stages is a major scientific challenge in AD research.

Changes in synaptic function, the extent of synaptic loss, and the severity of cognitive impairment in patients are well-correlated (Robinson et al., 2014; Siedlecki-Wullich et al., 2019). Synaptic loss constitutes the pathological basis of early mild cognitive impairment (MCI) in AD and it is also the structural basis of AD dementia. Therefore, the role of synaptic lesions in the pathogenesis of AD has been increasingly investigated. Synaptic dysfunction is an early pathological change in the pathogenesis of AD. Moreover, synaptic neurotransmitter dysfunction occurs before β-amyloid plaque formation (Verges et al., 2011; Zhang et al., 2012; Dinamarca et al., 2019). Pathological changes in the early stages of AD include reversible functional changes such as the down-regulation of synaptic function (Scheff et al., 2006); the late stages of AD are characterized by irreversible degenerative changes and structural changes in the brain (Hsiao et al., 1996).

The pathogenesis of AD is extremely complex. synaptic dysfunctions, structural abnormalities, and synaptic loss can affect neural signaling, which in turn results in functional abnormalities in the neural network. Autopsy findings indicate that the synaptic function is low in the brains of patients with AD, and that different brain areas are closely involved in the synaptic function of abnormal protein expression (Robinson et al., 2014). There are many proteins closely related to synaptic function, such as Calsyntenin-1, GluR4, PSD95, and synaptic vesicle glycoprotein 2A (SV2A) (Mokin and Keifer, 2004; Clarke et al., 2019; Zhu et al., 2020). The SV2A is located in the presynaptic vesicle membrane of virtually all synapses (Bajjalieh et al., 1994). Therefore, SV2A can be used as a target for measuring synaptic density (Finnema et al., 2016). SV2A PET could provide a way to measure synaptic density quantitatively in living humans and to track changes in synaptic density with disease. It belongs to the major facilitator superfamily of transporter proteins and constitutes the membrane of synaptic vesicles. It is involved in the transport of synaptic vesicles, exocytosis and the release of neurotransmitters (Mendoza-Torreblanca et al., 2019). SV2A plays an important role in maintaining normal neurotransmission. The absence of SV2A resulted in the decrease of gamma-Aminobutyric acid (GABA) neurotransmission in the hippocampus of mice, and there was evidence that SV2A dysfunction impaired GABA release and induced seizures (Crowder et al., 1999; Tokudome et al., 2016). As a specific binding target of various antiepileptic drugs, *in vivo*, synaptic density imaging targeting SV2A has been used in patients with temporal lobe epilepsy (Finnema et al., 2020). The decrease of synaptic number is also the pathological manifestation of AD patients. Preclinical genetic studies and those examined changes in protein expression have shown that SV2A dysfunction plays an important role in the pathogenesis of AD (Löscher et al., 2016). SV2A is more evenly distributed in the vesicles, therefore, assessing SV2A expression levels can more accurately measure the synaptic density. Moreover, the development of drugs that target SV2A may promote the progress of early diagnosis and treatment of AD. Previous studies have shown that Fyn inhibitors can reduce the loss of synaptic density and alleviate memory impairment in AD mice (Kaufman et al., 2015).

In this brief review, we summarized the involvements of SV2A in relation to tau hyperphosphorylation and β-amyloid plaque formation, and speculated the potential mechanism of SV2A in the occurrence and development of AD. At the same time, we introduced the development of SV2A PET imaging agents in recent years and analyzed the characteristics of the present imaging agents. Finally, we emphasize the advantages of PET imaging targeting SV2A in the early diagnosis of AD.

## Calcium Ion Regulates Synaptic Vesicle Glycoprotein 2A Mediated tau Hyperphosphorylation

One of the most typical pathological features of Alzheimer’s disease is neurofibrillary tangles (NFTs), which result from hyperphosphorylated tau protein (Crystal et al., 1988). In general, tau protein is beneficial to maintain the normal function of neurons, but the learning and memory ability will be impaired after tau hyperphosphorylation, and synaptic loss will be induced. These pathological changes often occur before the formation of neurofibrillary tangles (Boekhoorn et al., 2006; Yoshiyama et al., 2007; Kimura et al., 2010). We speculate that the deletion of SV2A may lead to the increase of tau protein hyperphosphorylation, resulting in synaptic loss and neurofibrillary tangles.

The degree of cognitive decline closely relates to the loss of synaptic function in the limbic system and neocortex (Terry et al., 1991). The degeneration of neurons is one of the causes of synaptic loss in AD. Hyperphosphorylated tau protein can induce cognitive impairment in the brain. The study has confirmed that there are two mechanisms of this induction in the mouse model of human tau pathology: low level of phosphorylated tau expression causes synaptic dysfunction, while the high level of phosphorylated tau promotes high activation of astrocytes and eventually leads to neuron loss (Di et al., 2016). In a correlational study with other pathological changes of AD, synaptic loss and NFTs were strongly associated with each other (Merino-Serrais et al., 2013). Abnormal phosphorylation of tau protein was detected in the synaptic terminals of the brain in patients with AD and AD mouse models. This provided a direct link between tau hyperphosphorylation and synaptic pathology.

It has been reported that SV2A density in AD is negatively correlated with the increase of tau phosphorylation, suggesting that SV2A can regulate the occurrence and development of AD (Metaxas et al., 2019). Dysregulation of SV2A expression and tau protein phosphorylation can cause synaptic dysfunction. Research has shown that tau phosphorylation mediates the synaptic abnormality caused by tau misplacement and the accumulation of dendritic spines, which disrupts synaptic function and causes synaptic damage by impairing glutamate receptors and N-methyl-D-aspartate receptor (NMDAR) transport (Hoover et al., 2010). In addition, SV2A dysfunction can activate NMDAR, and cause calcium overload and continue to generate action potentials (Tokudome et al., 2016). Tau–tubulin protein kinases are associated with SV2A and perform synaptic functions (Zhang et al., 2015). Hyperphosphorylated tau affects presynaptic function through its n-terminal structural domain and synaptic vesicle node, reducing synaptic vesicle and neurotransmitter release and reducing nerve signal transmission. Therefore, we speculated that SV2A regulates hyperphosphorylated tau protein by regulating the release of presynaptic calcium and neurotransmitters to reduce its content and affect AD.

## Synaptic Vesicle Glycoprotein 2A Participates in the Cleavage of App and the Accumulation of Reactive Oxygen Species Through Calcium-Related Pathways

Studies have shown that there is a particularly strong correlation between Aβ oligomers (AβOs) and loss of synaptic plasticity, as well as the consequent disorders. AβOs affect synaptic function and structural integrity. The accumulation of AβOs causes synaptic loss, which is the leading cause of learning and memory impairment, and ultimately leads to neuronal death. However, limited methods are available for the quantitative evaluation of AβOs, and the specific mechanism underlying AβOs accumulation induced synaptic damage remains unclear.

The decrease of synaptic plasticity caused by AβOs is related to the downregulation of SV2A expression. Confocal microscopy examinations revealed a reduced or absent SV2A immunoreactivity in the vicinity of neurons with AβOs aggregation in cerebral palsy and the frontal cortex in AD, providing direct morphological evidence for the correlation between AβOs aggregation and synaptic loss. The effect of AβOs on synaptic plasticity is closely related to mitochondrial dysfunction (McInnes, 2013). More and more evidence shows that Aβ can cause abnormalities in calcium homeostasis and increased reactive oxygen species (ROS), which leads to mitochondrial dysfunction. It has been shown that SV2A is also a mitochondrial protein and that Aβ is co-located with mitochondria (Stockburger et al., 2016; Takada et al., 2020). We speculate that SV2A may play a potential role in the oxidative stress induced by Aβ. This finding further supports the notion that AβOs aggregation is closely related to synaptic failure and might be the main cause of decreased cognitive function in patients with AD. AβOs aggregation can also inhibit long-term hippocampal potentiation and disrupt synaptic plasticity, both of which are closely related to memory loss in transgenic animal models of AD.

SV2A may be involved in the cleavage of AβPP by β-secretase through the calcium-related pathway. A study has shown that the cleavage of AβPP by α-secretase and β- secretase may occur in synaptic vesicles (Lundgren et al., 2015). This suggests that SV2A may interact with beta-site amyloid precursor protein cleaving enzyme 1 (BACE1) to affect the production of Aβ. Experimental studies have shown that synaptic activity can mediate the neuronal production and release of Aβ, and increased synaptic activity can increase Aβ secretion, which consequently can initiate a feedback loop that inhibits synaptic transmission. Changes in synaptic activity caused by abnormal synaptic vesicle formation might be another factor affecting the normal feedback loop, or might constitute the morphological basis between degenerated nerve endings and extracellular SP. The interaction between SV2 and synaptotagmin is mainly dependent on SV2A and is inhibited by calcium (Schivell et al., 1996). FE65, also known as APP-binding family B member 1 (APBB1), is a brain-enriched phospho- adaptor protein that interacts with APP and has been shown to modulate APP processing in AD. Phosphorylation of FE65 enhances β-secretase-mediated AβPP processing and the release of Aβ (Lee et al., 2017). SV2A interacts with the sarcoplasmic/endoplasmic reticulum calcium ATPase 2 (SERCA2) to regulate the calcium homeostasis of cells and is involved in the metabolism of AβA4 precursor protein-binding family B member 1, FE65 (Nensa et al., 2014). Therefore, we speculate that SV2A may be involved in AD through an intracellular calcium-related pathway.

## Synaptic Vesicle Glycoprotein 2A Affects Synaptic Transmission Through Calcium Channels

**FIGURE 1:**
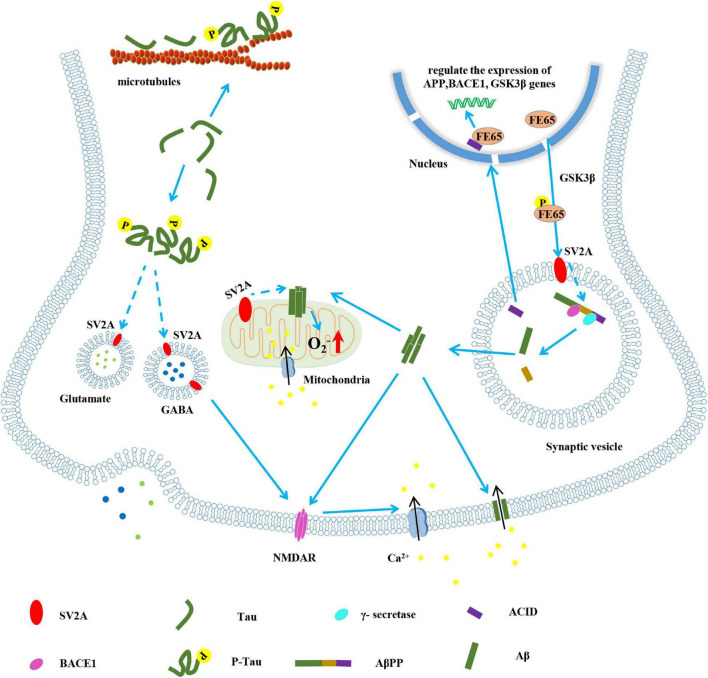
The pathological role of SV2A in the pathogenesis of AD. Known (solid lines) and hypothetical (dotted lines).

SV2A influences the concentration of calcium ions and ATP by regulating synaptic vesicle exocytosis and the release of the calcium-binding protein synaptophysin (SYN) (Wan et al., 2010; Casillas-Espinosa et al., 2012). SV2A knockout mice showed a decrease in the evoked action potentials of inhibitory neurons. Concomitantly, the release of calcium-dependent synaptic neurotransmitters is increased. That is, SV2A regulates the release of presynaptic calcium ions, and its dysfunction can activate NMDAR, resulting in calcium ion aggregation and continued action potentials, which ultimately leads to increased release of excitatory neurotransmitters and instability of the neural circuits (Figure 1; Casillas-Espinosa et al., 2012).

AβPP was cleaved to Aβ and intracellular AβPP domain (ACID) by BACE1 and γ-secretase. The oligomeric Aβ (1–42) can be inserted into the cell membrane to form a calcium channel, thereby causing calcium influx (Bode et al., 2017). ACID is also generated during the formation of Aβ, which forms a complex with FE65 to regulate the expression of genes such as AβPP, BACE1 and glycogen synthase kinase-3β (GSK3β) in the nucleus; FE65 is phosphorylated under the action of GSK3β. Phosphorylated FE65 can promote the cleavage of AβPP by BACE1 and γ-secretase. It is speculated that this may depend on the interaction between SV2A and phosphorylated FE65 (Nakaya and Suzuki, 2006; Nensa et al., 2014). Aβ acts on mitochondria, causing calcium influx and production of oxygen free radicals (Stockburger et al., 2016). SV2A, which is located on mitochondria, may be involved in this process. On the one hand, tau hyperphosphorylation leads to the weakening of microtubule stability and the disintegration of microtubule (Kadavath et al., 2015); on the other hand, it is speculated that phosphorylated tau interacts with SV2A to affect the movement and release rate of synaptic vesicles, and cause the abnormal secretion of GABA and glutamate neurotransmitters (Zhou et al., 2017; Vanoye-Carlo and Gómez-Lira, 2019). The abnormal function of SV2A also activates NMDAR, which makes calcium ions accumulate and produces action potential (Custer et al., 2006). The formation of Aβ oligomer, the oxidative stress of mitochondria and the instability of neural circuits all may cause synaptic damage, which may lead to AD.

Differential co-expression analysis has shown that SV2A is involved in the metabolism of Aβ/AβPP and may play the role of a “node” or “main regulator” in late-onset AD (Najm et al., 2019). A prospective, case-control study showed that patients with AD often have comorbid epilepsy. After analyzing 20745 individuals with comprehensive clinical data, it was found that the risk of seizures increases with the development of the course of AD patients (Vöglein et al., 2020). SV2A plays an important role in the pathophysiology of epilepsy, and its ligand may be closely related to the treatment of epilepsy and AD (Löscher et al., 2016). Levetiracetam (LEV) selectively targets SV2A and attenuates excitatory neurotoxicity by inhibiting voltage-dependent calcium channels, thereby conferring neuroprotection (Vogl et al., 2015). In addition to improving epilepsy, as compared to the control group, LEV significantly improved cognition, short-term memory, and language expression in an experimental cohort of patients with AD (Cumbo and Ligori, 2010). As an SV2A ligand, UCB0255 has no anticonvulsant properties, but it has high selectivity and affinity for SV2A (p*K*_i_ = 7.9), and improves cognitive abilities in animals models of AD (Laruelle et al., 2014). Therefore, we speculate that SV2A is involved in the regulation of synaptic calcium channel signaling, which is related to the occurrence of AD. Combined with molecular imaging, the development of imaging agents targeting SV2A based on the high-affinity ligand of SV2A may become an effective method for quantitative analysis of synaptic density and early diagnosis of AD.

## Development Progress of Synaptic Vesicle Glycoprotein 2A Imaging Agent

Presynaptic neuronal activity is initiated by numerous synaptic vesicles containing neurotransmitters, which is a diffusible chemical signal. These changes in synaptic function are closely related to the pathogenesis of AD. However, the current analysis and quantification of synaptic density require surgery and anatomy to obtain brain tissue. Both these methods have limited clinical applications (Finnema et al., 2016). Therefore, it is extremely important to develop a non-invasive analytical method for *in vivo* quantification of synaptic density (Buckley and Kelly, 1985). As a structural component of the presynaptic membrane and synaptic vesicles, the detection of SV2A can better indicate neural plasticity following an injury. In other words, SV2A PET analysis can be served as a functional marker of synapses (Table 1).

**TABLE 1:** SV2A positron emission computed tomography (PET) imaging.

**Tracer**	**Human**	**Animal model**	**Main findings**	**References**
UCB-H		C57BL-6 mice, OFA rats	[^18^F]UCB-H is the first reported PET tracer for SV2A. The effective dose (1.86E-02 mSv/MBq) of [^18^F]UCB-H meets the standard criteria for radiation exposure in clinical studies.	Bretin et al., 2013; Warnock et al., 2014
	Healthy male volunteers		This first human dosimetry study of [^18^F]UCB-H indicated that the tracer shows similar radiation burdens to widely used common clinical tracers. However, [^18^F]UCB-H provides limited specific binding signals in monkeys and humans.	Bretin et al., 2015; Bahri et al., 2017
[^11^C]-levetiracetam ([^11^C]-LEV)			The multistep preparation of [^11^C]-LEV was carried out by a one-pot radiosynthesis with 8.3 ± 1.6% radiochemical yield in 50 ± 5.0 min. The radiochemical and enantiomeric purities of [^11^C]-LEV were > 98%.	Cai et al., 2014
[^11^C]UCB-A		SD rats; Pigs; APPswe/PS1δE	Radiochemistry and preclinical studies of [^11^C]UCB-A in rats and pigs. [^11^C]UCB-A can be used to compare SV2A expression between individuals. However, [^11^C]UCB-A pharmacokinetics is slow.	Mercier et al., 2014; Estrada et al., 2016; Toyonaga et al., 2019
[^18^F]SynVes-2		Rhesus monkeys	[^18^F]SynVes-2 exhibited excellent image quality and suitable pharmacokinetics for quantitative kinetic modeling and provided high specific binding in non-human primates and humans. As a measure of specific binding, mean *BP*_ND_ values of [^18^F]SynVesT-2 were 42% lower than those of [^18^F]SynVesT-1, and 24% lower than those of [^11^C]UCB-J, but threefold higher than those of [^18^F]UCB-H.	Cai et al., 2020a, b
(R)-^18^F-SDM-7		Non-human primates	(R)-^18^F-SDM-7 has attractive imaging properties: high brain uptake, fast tissue kinetics, and high specific-to-non-specific binding ratios in brain. *BP*_ND_ values of (R)-^18^F-SDM-7 are comparable to ^11^C/^18^F-UCB-J and much higher than ^18^F-UCB-H.	Cai et al., 2018
	Healthy human, epilepsy subjects, AD and CN participants		[^11^C]UCB-J demonstrated excellent PET tracer characteristics and has potential for measuring synaptic density. First-in-human PET studies demonstrated that [^11^C]UCB-J had excellent imaging properties and was sensitive to synaptic loss in patients with temporal lobe epilepsy. [^11^C]UCB-J could effectively evaluate SV2A occupancy and human brain penetration time course of different treatment drugs at therapeutically relevant doses. There were widespread reductions of SV2A binding in medial temporal and neocortical brain regions in early AD compared to CN participants. Despite the excellent imaging properties of [^11^C]UCB-J, the short radioactive half-life (20.4 min) places some restrictions on its broader application.	Finnema et al., 2016, 2018, 2019; Mecca et al., 2020
[^18^F]-UCB-J		Rhesus monkeys	The radiochemical yield of [^18^F]-UCB-J is low. [^18^F]-UCB-J displayed pharmacokinetic and imaging characteristics similar to those of [^11^C]UCB-J, with moderate metabolism rate, high brain uptake, fast and reversible binding kinetics, and high specific binding signals in non-human primates.	Li et al., 2019b
[^18^F]SynVesT-1 (or [^18^F]-MNI-1126, [^18^F]-SDM-8)	Healthy volunteers, AD, PD		[^18^F]SynVesT-1 has similar pharmacokinetic characteristics with [^11^C]UCB-J and has a longer half-life (108 min). [^18^F]SynVesT-1 demonstrates outstanding characteristics in humans: high brain uptake, fast and reversible kinetics, high levels of specific binding (mean *BP*_ND_ values were 21% higher than [^11^C]UCB-J), and excellent test-retest reproducibility of binding parameters. The SV2A density is decreased in specific regions in PD and AD subjects.	Chen et al., 2018; Li et al., 2019a, 2021; Constantinescu et al., 2019

Because of the wide distribution of SV2A in the brain and as a target of antiepileptic drug levetiracetam, the development of SV2A PET imaging agent has been started in 2013 (Bretin et al., 2013). [^18^F]UCB-H is the first developed SV2A imaging agent. It has high affinity and selectivity to SV2A *in vitro*, and it can be safely used in the human body in clinical dosimetry (Single injections of at maximum 672 MBq for US practice and 649 MBq for European practice keep radiation exposure below recommended limits.). However, it is difficult to obtain [^18^F]UCB-H by a four-step radiosynthesis process (Bretin et al., 2013, 2015; Warnock et al., 2014). Based on LEV, Hancheng Cai et al. (2014) developed [^11^C] levetiracetam ([^11^C]-LEV), but its clinical application is limited due to the moderate binding affinity of LEV and SV2A. *In vivo* studies have found that Brivaracetam (BRV) is an SV2A ligand with higher affinity than LEV, as well as higher brain permeability and faster SV2A occupancy. BRV has more advantages than LEV in the treatment of epilepsy, but there is no SV2A PET tracer developed based on BRV (Nicolas et al., 2016; Finnema et al., 2019). Based on the 3D pharmacophore model of levetiracetam, Mercier et al. (2014) reported the rationale design of three heterocyclic non-acetamide lead compounds, UCB-A, UCB-H and UCB-J. UCB-J, a LEV analog that selectively acts on the major transmembrane hydrophilic residues of SV2A (T456, S665, W666, D670, and L689 residues), can be used (Correa-Basurto et al., 2015). Synaptic density can be quantitatively analyzed *in vivo* by PET using [^11^C]UCB-J. A few years later, two imaging agents, [^11^C]UCB-J and [^11^C]UCB-A, were developed (Estrada et al., 2016; Nabulsi et al., 2016). In humans, [^11^C]UCB-J PET has excellent imaging characteristics and can be used for quantitative estimation of synaptic density, which has significant potential for use in the early diagnosis and evaluation of AD (Finnema et al., 2016; Nabulsi et al., 2016; Toyonaga et al., 2019). Ming-Kai Chen et al. (2018) first demonstrated that synaptic density was reduced in the hippocampus of living people with AD, using PET imaging of SV2A with the radioligand [^11^C]UCB-J. Nabulsi et al. (2016) performed [^11^C]UCB-J imaging on 21 subjects (10 AD patients and 11 normal subjects) and showed that the average binding potential (*BP*_ND_) in the hippocampus of the normal group was 1.47, and the average *BP*_ND_ of the hippocampus in the mild AD group was 0.87, indicating that the binding of [^11^C]UCB-J in the hippocampus of patients with mild AD was significantly lower than that of the normal group. In addition, synaptic density was measured in 34 patients with AD using [^11^C]UCB-J PET. It was found that SV2A binding in the hippocampus and entorhinal cortex decreased significantly (Mecca et al., 2020). This result is consistent with the autopsy results, which showed that the outer synapses of the dentate gyrus in the hippocampus of AD and MCI patients were reduced by 13–44% (Masliah et al., 1994; Scheff et al., 2006). In addition, an autopsy immunohistochemical study of 157 subjects (84 patients with AD, 37 patients with MCI, and 36 normal subjects) showed that the expression of SV2A in the hippocampus of AD and MCI groups was significantly lower than Normal cognition group (Robinson et al., 2014). It can be seen that the expression level of SV2A in AD patients is characteristically lower in the hippocampus compared with normal people. SV2A-PET imaging can directly reflect the synaptic density in the hippocampus of AD patients, and its imaging characteristics are consistent with the pathological characteristics of AD. It has good specificity and can dynamically assess the patient synaptic loss situation, monitoring disease progression and therapeutic intervention in patients with a better assessment.

Brain dynamics studies in rats and pigs have shown that [^11^C]UCB-A pharmacokinetics is slow (Estrada et al., 2016). Although [^11^C]UCB-J has excellent PET tracer properties in kinetic evaluation, the short radioactive half-life (20.4 min) has limited its clinical application. In contrast, ^18^F has an optimal half-life (108 min) and lower mean positron energy than ^11^C (^11^C: 970 keV and ^18^F:635 keV), and is suitable for the preparation of PET tracers. Therefore it is a better positron imaging nuclide. The best method of PET imaging involves performing structural improvements to [^11^C]UCB-J without changing its activity, and thereafter, performing F-labeling to synthesize [^18^F]UCB-J. But there is a challenge in the synthesis of [^18^F]UCB-J. Researchers tried to synthesize [^18^F]UCB-J based on chlorine/iodine precursors, boron, iodonium, matte or tin precursors. The products with [^18^F] labeling could not be synthesized by conventional methods but could be successfully prepared by using high valent iodonium precursors. In the rhesus monkey, it was found that [^18^F]UCB-J reached peak uptake in the whole brain region 30 min after injection, It has similar pharmacokinetic characteristics with [^11^C]UCB-J and has a longer half-life (110 min) (Li et al., 2019b). However, the lower synthetic yield of [^18^F]UCB-J has brought some limitations to its wide application, and its synthesis scheme and conditions need to be further optimized.

[^18^F]SDM-7 is the ^18^F labeled SV2A tracer developed by Cai et al. (2018) team of the Radiology and Biomedical Imaging Department of Yale University in previous years. *In vivo* imaging evaluation showed that [^18^F]SDM-7 had high brain uptake and fast tissue dynamics (brain activity-to-AIF ratios plateauing after 20 min for [^18^F]SDM-7, vs. 50 min for [^11^C] UCB-J or [^18^F]UCB-J). But its low affinity (*K*_i_ of 2.1 nM and 9.4 nM for the (*R*)- and (*S*)-enantiomer of SDM-7, vs. *K*_i_ of 0.27 nM for UCB-J) might limit its development (Cai et al., 2018). [^18^F]SynVesT-1 is a newly developed SV2A PET tracer by Li et al. (2019a), also known as [^18^F]SynVesT-1, belonging to [^18^F] labeled UCB-J two fluorine analogs. Compared with [^18^F]SDM-7, [^18^F]SynVesT-1 is a better SV2A tracer. *In vivo* evaluation of rhesus monkeys showed that [^18^F]SynVesT-1 had higher uptake (peak SUV of 8) in the brain and higher molar activity (241.7 MBq/nmol) with SV2A (Li et al., 2019a). The SV2A density measured by [^18^F]SynVesT-1 in human studies showed that the largest reductions in *BP*_ND_ were found in hippocampus (51%), and superior lateral temporal cortex (39%) in AD. Meanwhile, the largest reductions in *BP*_ND_ were found in caudate nucleus (38%), hippocampus (33%), and thalamus (30%) in PD (Constantinescu et al., 2019).

All these indicators suggested that [^18^F]SynVesT-1 was a potential radioactive tracer for SV2A. Then the research team evaluated the imaging effect of [^18^F]SynVesT-1 in the human body. The metabolic rate of [^18^F]SynVesT-1 and [^11^C] UCB-J was measured at 10 and 30 min after injection, and it was found that the kinetic difference between the two was not obvious, but in terms of binding characteristics, [^18^F]SynVesT-1 average *BP*_ND_ value of the brain is 21% higher than that of [^11^C] UCB-J. In addition, the kinetic parameters and specific binding parameters of [^18^F]SynVesT-1 have excellent retest reproducibility. Therefore, [^18^F]SynVesT-1 is expected to be an excellent SV2A tracer for clinical imaging and quantification of humans (Li et al., 2019c, 2021; Naganawa et al., 2020). In addition, Cai et al. (2020a, b) team synthesized a new ^18^F -labeled candidate SV2A imaging probe [^18^F] SynVesT-2. *In vivo* evaluation of rhesus monkeys, this probe has faster dynamics than [^11^C] UCB-J and [^18^F]SynVesT-1, while human evaluation is still in progress.

The discovery and establishment of a simple and effective diagnostic marker that is closely related to functional outcomes is an important goal in current AD research. Synaptic loss is the main cause of cognitive impairment in AD. As a membrane protein on synaptic vesicles, SV2A is a good target for representing synaptic damage. The lack of an optimal predictive biomarker has become a key scientific issue in the research and clinical practice of AD. SV2A, in combination with the currently recognized AD model (involving the formation and accumulation of SPs and NFTs as the pathological mechanism), can be used as a marker of synaptic alteration. Molecular imaging, behavioral and molecular biology and morphological methods are used to observe the effects of SV2A in the pathogenesis of AD. Moreover, SV2A can be used to further identify the possible mechanisms underlying synaptic dysfunction and loss in AD, and it can also be used as a new target for the early diagnosis and treatment of AD (Stockburger et al., 2016).

## Application Synaptic Vesicle Glycoprotein 2A Positron Emission Computed Tomography in the Early Diagnosis of Alzheimer’s Disease

The dysfunction of synaptic function and the decrease of synaptic density are the typical pathological features of AD. Therefore, SV2A, a protein commonly expressed in synaptic vesicles, can be used as a biomarker for measuring synaptic density. Data show that three of the fifty-seven Alzheimer’s disease-related clinical trials funded by the National Institute on Aging are related to SV2A, and the study of LEV and AGB101 (levetiracetam antagonist) has entered phase II/III trials, which suggests that SV2A is a potential marker for detecting synaptic density. However, the current measurement of synaptic density in neurodegenerative diseases is still focused on the detection of synaptic protein levels in cerebrospinal fluid (Mazzucchi et al., 2020). With the development of PET technology, [^18^F]-FDG has realized the accurate detection of synaptic function, and SV2A PET provides an indicator for the direct measurement of synaptic density. [^11^C]UCB-J is the main clinical SV2A tracer. New studies have confirmed that volume of distribution (*V*_T_) and *BP*_ND_ of [^11^C]UCB-J are stable when measuring synaptic density, because they are not affected by changes in tracer concentration caused by increased neuronal discharge during physiological brain activation (Smart et al., 2020). This distinguishing feature of [^11^C]UCB-J greatly promotes its wide clinical application. The team of Yale School of Medicine used [^11^C]UCB-J for the first time to measure the synaptic density of AD patients, which directly confirmed that there is a significant decrease in synaptic density in the hippocampus of AD patients (Chen et al., 2018). In subsequent clinical trials, the team used [^11^C]UCB -J to perform PET imaging of SV2A to further confirm that there is a decrease in synaptic density in the medial temporal area and neocortical brain area of early AD patients (Mecca et al., 2020). To determine the association between Aβ deposition and synaptic loss, researchers used [^11^C]PIB PET and [^11^C]UCB-J PET to measure Aβ deposition and synaptic density in patients with AD-induced mild cognitive impairment and mild dementia. The results found that Aβ deposition in the brain of patients with mild cognitive impairment is negatively correlated with synaptic density, which indicates that Aβ is closely related to synaptic loss in the early stages of AD (O’Dell et al., 2021). Further study found that the early cognitive ability of AD patients also was significantly associated with synaptic density (Sharp et al., 2021). [^18^F]FDG PET has its limitations. Because brain [^18^F] FDG uptake is often affected by sensory stimulation, medications, fasting status, and blood glucose. These limitations in precision may ultimately hinder accurate assessment of disease progression or therapeutic effects in longitudinal clinical trials using [^18^F]FDG PET. [^11^C]UCB-J binding and [^18^F]FDG metabolism showed a similar magnitude of reduction in the medial temporal lobe of AD compared to CN participants. However, the magnitude of reduction of [^11^C]UCB-J binding in neocortical regions was less than that observed with [^18^F]FDG metabolism. Inter-tracer correlations were also higher in the medial temporal regions between synaptic density and metabolism, with lower correlations in neocortical regions. [^11^C]UCB-J perfusion showed a similar pattern to [^18^F]FDG metabolism, with high inter-tracer regional correlations. The first *in vivo* PET imaging of synaptic density and metabolism in the same AD participants and reported a concordant reduction in medial temporal regions but a discordant reduction in neocortical regions (Chen et al., 2021). Compared to healthy controls, patients with aMCI have synaptic density loss indicating by [^11^C]UCB-J binding mainly in medial temporal lobe (MTL), which is known to be involved in early cognitive impairment. Furthermore, increased tau deposition indicating by ^18^F- MK6240 binding in the same region (Vanhaute et al., 2020). The latest research shows that Tau pathology is closely associated with affected synaptic density and synaptic function in Alzheimer’s disease, which is verified by [^18^F]flortaucipir PET), [^11^C]UCB-J PET and magnetoencephalography (MEG) in Alzheimer’s disease (Coomans et al., 2021).

## Discussion

β-amyloid plaque deposition and neurofibrillary tangles are the most typical pathological features of Alzheimer’s disease. In the early stage of these pathological changes, synaptic loss and synaptic dysfunction have occurred, which are the key factors causing cognitive impairment factors. SV2A is a synaptic vesicle protein, which plays an important role in maintaining the normal synaptic function, such as the release of neurotransmitters. More and more studies indicate that SV2A may be a key molecule in the development of Alzheimer’s disease. On the one hand, SV2A immune response is absent near the neurons with AβOs aggregation or based on the influence on synaptic activity, SV2A interacts with FE65, SERCA2, BACE1, and participates in the production of Aβ through the calcium-related pathway, thus inducing AD. On the other hand, SV2A may be involved in tau phosphorylation by regulating the release of calcium ions and neurotransmitters, which leads to nerve fiber degeneration and finally to AD.

SV2A can quantify the density of synapses, relying on PET technology, choosing the right SV2A imaging agent can diagnose early AD. These excellent imaging agents such as [^11^C]UCB-J, [^18^F]SynVesT-1 and [^18^F]SynVesT-2 are in clinical trials or have been used in clinical trials. They display excellent kinetic and *in vivo* binding properties in animal models or humans and hold great potential for the imaging and quantification of synaptic density in patients with AD. China has a large and increasingly aging population. The social, familial, and medical burdens caused by AD will become increasingly severe over time. Therefore, important theoretical innovations and the development of independent intellectual property rights in AD research can greatly improve the level of AD research in China and promote the development of improvements in the clinical diagnosis and treatment of AD.

## Author Contributions

YK, YG, BS, and JW contributed to the conception and design of the review. YK wrote the first draft of the manuscript. SZ, LH, CZ, FX, ZZ, QH, DJ, JL, WZ, and TH wrote sections of the manuscript. YK revised the manuscript and approved the final version. YK and SZ prepared the figure and table. All authors contributed to manuscript revision, read, and approved the submitted version.

## Conflict of Interest

The authors declare that the research was conducted in the absence of any commercial or financial relationships that could be construed as a potential conflict of interest.

## Publisher’s Note

All claims expressed in this article are solely those of the authors and do not necessarily represent those of their affiliated organizations, or those of the publisher, the editors and the reviewers. Any product that may be evaluated in this article, or claim that may be made by its manufacturer, is not guaranteed or endorsed by the publisher.

## Abbreviations

OFA: Oncins France strain A Sprague-Dawley
AD: Alzheimer’s disease
SV2A: synaptic vesicle glycoprotein 2A
C57BL-6: C57 black 6
LEV: levetiracetam
SD: Sprague–Dawley
PET: Positron Emission Computed Tomography
CN: cognitively normal
Aβ: β-amyloid
MCI: mild cognitive impairment
NFTs: neurofibrillary tangles
NMDAR: N-methyl -D-aspartate receptor
A β Os: A β oligomers
ROS: reactive oxygen species
GABA: gamma-Aminobutyric acid
BACE1: beta-site amyloid precursor protein cleaving enzyme 1
APBB1: APP-binding family B member 1
SERCA2: sarcoplasmic/endoplasmic reticulum calcium ATPase 2
BRV: Brivaracetam
MTL: medial temporal lobe
MEG: magnetoencephalography.
